# Large-Scale Evaluation of IgM and IgG Lateral Flow Assays for Rapid Diagnosis of Human Brucellosis

**DOI:** 10.3390/diagnostics16213500

**Published:** 2026-10-26

**Authors:** Rajeswari Shome, Somy Skariah, Gandu Shanmugam, Nanditha T. Rajeshwari, Pavan K. Nagaraja, Snigdha M. Maharana, Arijit Shome, Duralb P. Bora, Nagalingam Mohandoss, Nagendra N. Barman

**Affiliations:** 1 Indian Council of Agricultural Research-National Institute of Veterinary Epidemiology and Disease Informatics (ICAR-NIVEDI), Bengaluru 560119, India; 2 College of Veterinary Science, Assam Agricultural University, Khanapara, Guwahati 781022, India

**Keywords:** brucellosis, IgM_LFA, IgG_LFA, RBPT, SAT, veterinary healthcare personnel

## Abstract

**Background:** Human brucellosis is a major occupational hazard in India, especially among veterinary healthcare workers due to frequent contact with livestock. This study evaluated in-house standardized IgM and IgG-based lateral flow assays (LFAs) against a Rose Bengal Plate Test (RBPT) and a Serum Agglutination Test (SAT). **Methods:** A total of 1356 serum samples (392 veterinary doctors and 964 paraveterinary staff) were sourced for evaluation of the tests across Karnataka, India. The results were analyzed for performance metrics {DSe (Diagnostic Sensitivity), DSp (Diagnostic Specificity), PPV (Positive Predictive Value), NPV (Negative Predictive Value), accuracy, and kappa with 95% CI} through chi-square and regression analyses. **Results:** Overall seropositivity was 7.67% (104/1356; 95% CI: 6.31–9.22) by RBPT, 3.47% (47/1356; 95% CI: 2.56–4.58) by SAT, 2.95% (40/1356; 95% CI: 2.12–4.00) by IgM_LFA, and 6.34% (86/1356; 95% CI: 5.10–7.77) by IgG_LFA. The RBPT vs. IgM and IgG_LFA showed a DSe of 85.3% (74.6–92.7) and 82.69% (74.03–89.41), respectively. Compared with SAT, IgM_LFA and IgG_LFA demonstrated high DSe values of 95.35% (84.2–99.4) and 96.55% (88.1–99.6), respectively. Seroprevalence was higher among paraveterinary staff (8.71%, CI 95%: 7.20–10.90) than veterinary doctors (5.10%, CI 95%: 3.14–7.77), higher in males (8.30%, CI 95%: 6.77–10.04) than females (4.02%, CI 95%: 2.44–9.05) and higher in individuals aged >55 years (9.09%, CI 95%: 5.64–13.69) compared to other age groups. Regression analysis yielded coefficients of 62.90% (*p* = 0.0107) for veterinarians and 87.89% (*p* = 0.0001) for paraveterinarians. **Conclusions:** This integrated field evaluation confirms improvement in brucellosis detection and emphasizes the need for field-applicable diagnostic tools.

## 1. Introduction

Brucellosis is one of the most common zoonotic illnesses in the world. It is also referred to as Mediterranean fever, undulant fever, or Malta fever, causing significant economic losses in livestock globally [1]. More than 160 nations in the Mediterranean region, Africa, South and Central America, Asia, the Arabian Peninsula, the Indian subcontinent, and the Middle East still have endemic cases of the illness, with a wide range of human incidence from 0.01 to over 200 cases per 100,000 population [2,3,4,5,6]. Brucellosis is closely linked to animal infections, putting certain high-risk occupational groups such as farmers, veterinarians, butchers, dairy workers, animal handlers, meat inspectors, and laboratory staff at greater risk. The disease primarily spreads through direct contact with infected animals or by consuming unpasteurized milk and dairy products. The disease causes chronic, crippling illness and significantly increases disability-adjusted life years (DALYs), with occupational populations being disproportionately affected [6,7].

In India, human brucellosis represents a major occupational hazard for farmers, animal handlers and animal healthcare workers where approximately 70% of the human population living in rural areas are frequently interacting with livestock. National data shows fluctuating trends of human brucellosis from 1990 to 2025, with regional variations influenced by livestock density, dairy consumption patterns, and diagnostic capacity. Between 1990 and 2010, seroprevalence rates ranged from 1 to 20%, with higher incidences in rural areas due to close animal contact and consumption of raw milk [5]. States such as Punjab, Haryana, Rajasthan, and Gujarat, which have large livestock populations, consistently reported higher incidence rates [6,8]. Several studies have reported 2.26% to 34% prevalence of human brucellosis among veterinarians and paraveterinarians in high milk-producing regions of Northern, Western, and Southern India [9,10]. In endemic regions, brucellosis frequently presents as a significant cause of fever of unknown origin (FUO) cases [5,11,12,13].

The diagnosis of human brucellosis primarily depends on serological methods such as the Rose Bengal Plate Test (RBPT) and Serum Agglutination Test (SAT), though these tests are constrained by their limited sensitivity, particularly in chronic cases. Enzyme-linked immunosorbent assays (ELISAs) offer higher accuracy but require laboratory infrastructure and trained personnel, making them impractical for individual sample testing or limited samples. The RBPT and SAT antigens are locally available from government institutions in the country, whereas IgM and IgG ELISA kits need to be imported, adding substantial cost and procurement challenges.

Recently, lateral flow assays (LFAs) have emerged as rapid, point-of-care diagnostic tools suitable for field use for many infectious diseases, including brucellosis, elsewhere. Initial development and limited-sample evaluation of LFA kits in our laboratory showed satisfactory performance with IgM and IgG iELISA, suggesting their potential for broader application [8]. Further large-scale evaluation was deemed essential after comparison with routinely employed tests such as RBPT and SATs, in order to gain confidence and to validate their usefulness for translation into routine occupational health screening. To our knowledge, this study represents the first large-scale in-house developed evaluation of IgM and IgG_LFA for human brucellosis, aiming to provide a practical diagnostic tool for high-risk personnel such as veterinary staff and farmers.

## 2. Materials and Methods

### 2.1. Study Site and Population

The Indian Council of Agricultural Research-National Institute of Veterinary Epidemiology and Disease Informatics (ICAR-NIVEDI), Bengaluru, India, under the Ministry of Agriculture and Farmers Welfare, Government of India, has been conducting brucellosis surveys in livestock for three decades. The Institute has facilities to execute research on zoonotic diseases, including brucellosis, in a biosafety laboratory 2+ facility with special reference to surveillance and sero-monitoring of brucellosis in livestock population [14]. Routine requests are received from various medical and veterinary institutions, animal husbandry departments and veterinary healthcare staff for diagnosis of human brucellosis. For veterinary healthcare personnel, brucellosis awareness and screening camps were conducted in nine regions of Karnataka state, India (Figure 1). During such camps, qualified medical staff collected 4–5 mL blood samples in vacutainer tubes without anticoagulants (Becton, Dickinson U.K. Limited) from willing participants. Data on age, sex, type of employment, and livestock exposure history were collected using a standardized questionnaire for interpretation of disease status, along with informed consent for participation in the brucellosis study. The inclusion criteria comprised individuals employed in veterinary healthcare for more than one year, while those involved exclusively in administrative tasks without animal contact were excluded. In total, 1356 blood samples were sourced, consisting of 964 from paraveterinary staff and 392 from veterinary doctors, and were tested by RBPT and SAT, and their ability to provide a brucellosis diagnosis was examined, with the same set of samples utilized for evaluation of IgM and IgG_LFA (Figure 2).

### 2.2. Sample Processing and Testing

Serum samples were separated from blood clots collected in vacutainers and transported to ICAR-NIVEDI, Bengaluru, India, strictly maintaining the cold chain protocol. Upon receiving the serum samples, they were centrifuged at 500× *g* for three to five minutes and clear sera obtained from clots were coded and preserved at −20 °C until testing. The serum samples were tested for anti-*Brucella* antibodies using RBPT and SAT as per the defined protocols using positive and negative sera samples on each day of testing [15]. An antibody titer of ≥1:160 was considered significant for the diagnosis of human brucellosis [16]. For RBPT and SAT, both colored and plain *B. abortus* S99 antigens were procured from the Institute of Animal Health and Veterinary Biologicals (IAH&VB), Bengaluru, India.

All the samples were subjected to in-house developed IgM and IgG_LFA to detect anti-*Brucella* IgM and IgG antibodies. The test line of the LFA incorporated 100 ng of smooth lipopolysaccharide (sLPS) antigen from *B. abortus* S99 as the capturing antigen, while the control line used goat anti-mouse IgG antibody [8]. Anti-human IgM and IgG antibodies conjugated to 40 nm colloidal gold nanoparticles (Innova Biosciences, UK) were applied to the conjugate pad [8]. For each test, 10 µL each of serum sample was applied in two windows of fabricated LFAs followed by two to three drops of diluent buffer (1× phosphate-buffered saline with 1% Tween-20). Results were interpreted within one to three minutes, and the sample was considered positive for brucellosis if it showed a clear band in the IgM or IgG test cards of LFA. The following diagnostic criteria were followed to declare the seropositivity:

(a) Preliminarily RBPT positivity followed by a significant antibody titer 1:160 in SAT and/or
(b) Detection of both anti-*Brucella* IgM and IgG antibodies by IgM and IgG_LFA tests or sample positive by at least two serological tests.

### 2.3. Data Analysis

The serological test results were coded as binary (0 for seronegative, 1 for seropositive) to determine the statistical significance of variables and to analyze the relationship between host factors and the disease through a chi-square (χ^2^) test. The performance of the LFA was evaluated using MEDCALC statistical software 23.5.5. to calculate several diagnostic metrics including sensitivity (DSe), specificity (DSp), positive and negative predictive values (PPV/NPV), accuracy, and kappa statistics at a 95% confidence interval. Microsoft Excel 2021 was used to perform regression analysis, although a composite serological approach was used to define brucellosis positivity wherein RBPT and SAT were independently used as reference standards for comparative evaluation of IgM and IgG LFAs in order to align with routine diagnostic practices and assess the feasibility of LFAs as field-applicable alternatives. However, it is acknowledged that RBPT and SAT are imperfect reference standards and may influence the diagnostic performance outcomes.

## 3. Results

We sourced 1356 human serum samples from two risk personnel groups (paraveterinary staff71.09% and veterinary doctors28.90%) representing the age groups of <25, 25.1–35.0, 35.1–45.0, 45.1–55.0 and >55.1 years of both sexes [male-1157 (85.32%) and female-199 (14.67%)]. Overall seropositivity was 7.67% (104/1356; 95% CI: 6.31–9.22) by RBPT, 3.47% (47/1356; 95% CI: 2.56–4.58) by SAT, 2.95% (40/1356; 95% CI: 2.12–4.00) by IgM_LFA, and 6.34% (86/1356; 95% CI: 5.10–7.77) by IgG_LFA (Figure 3A,B). The highest seropositivity was recorded by RBPT [7.67% (104/1356)], of which, 47 cases [3.47% (2.56–4.58)] showed significant SAT titres. The distribution of cases in 1:160, 1:320, 1:640 and >640 SAT titres were 21 (44.68%), 13 (27.65%), 10 (21.27%) and 3 (6.4%), respectively. The highest seropositive results were observed for IgG_LFA with 86 positive cases (6.34%: CI 95%, 5.10–7.77)] compared to IgM_LFA with 40 positives (2.95%: CI 95%, 2.12–4.00) (Table 1).

The RBPT vs. IgM_LFA comparison showed a DSe of 85.30% (74.6–92.7) with excellent DSp of 100% (99.2–100), PPV of 100% (95.8–100), NPV of 99.55% (99.0–99.8), overall accuracy of 99.56% (99.0–99.8), and kappa value of 0.91 (0.85–0.96), indicating strong agreement between the assays. Similarly, RBPT versus IgG_LFA demonstrated a Dse of 82.69% (74.03–89.41), DSp of 100% (99.71–100), PPV of 100% (95.8–100), NPV of 98.58% (97.8–99.1), overall accuracy of 98.67% (97.9–99.2), and kappa value of 0.89 (0.85–0.94).

The SAT demonstrated excellent diagnostic performance compared to RBPT against both LFAs. Compared with IgM_LFA, SAT achieved a high Dse of 95.35% (84.2–99.4) along with perfect DSp of 100% (99.2–100), PPV of 100% (91.9–100), NPV of 99.84% (99.3–99.9), overall accuracy of 99.85% (99.4–99.9), and kappa value of 0.97 (0.94–1.00), indicating almost perfect agreement. Similarly, SAT versus IgG_LFA showed the highest Dse among all comparisons at 96.55% (88.1–99.6), coupled with DSp of 99.54% (98.36–99.9), PPV of 91.76% (73.6–97.8), NPV of 99.82% (99.3–99.9), overall accuracy of 99.41% (98.8–99.8), and kappa value of 0.96 (0.92–0.99) (Table 2; Figure 4A,B).

A greater number of samples was drawn for males [1157 (85.32%)] compared to females [199 (14.67%)] and the male-to-female ratio was 5.8:1. Out of 1157 male participants (85.32%), 96 were positive, giving a prevalence of 8.30% (CI 95%: 6.77–10.04). In comparison, among 199 females (14.67%), only eight tested positive, corresponding to 4.02% (CI 95%: 2.44–9.05). The chi-square test (χ^2^ = 3.80; *p* = 0.0051) indicated a significant association between sex and seropositivity, with males having more than twice the odds compared to females (OR = 2.16). When assessed by age groups, the highest prevalence was observed in individuals aged > 55 years (n-220, 16.22%), with 20 positives giving 9.09% (CI 95%: 5.64–13.69) (Table 3). A slightly higher prevalence was recorded in the 45.1–55 years group (n-447, 32.96%), where 36 showed seropositivity 8.05% (CI 95%: 5.70–10.98) and the 35.1–45 years group (n-302, 22.27%) showed moderate prevalence 7.95% (CI 95%: 5.16–11.59).

Regression analysis for all four serological tests yielded a coefficient of 62.90% (*p* = 0.0107) for veterinarians and 87.89% for paraveterinarians (*p* = 0.0001), indicating a moderate to high correlation between the number of samples and positive cases signifying statistical significance of the serological tests conducted in the study (<0.05) (Table 4; Figure 5A,B). Overall, paraveterinary staff had 4.05% significant SAT titres compared to 2.04% in veterinary doctors. Similarly, among 392 veterinary doctors tested, both IgM and IgG_LFA positives were low (1.78%) compared to 964 paraveterinary staff screened (3.21%). Individuals with titres 1:160 (320 IU/mL) were suggested to undergo further clinical correlation and treatment.

## 4. Discussion

Frequent nonspecific signs of human brucellosis include fever, sweating, and arthritis, which can mimic other infections. Untreated cases can progress to chronic stages with severe complications like endocarditis or neurological disorders [17,18]. Therefore, accurate differentiation between acute and chronic infections, along with periodic serological follow-up is essential for high-risk populations. Laboratory diagnosis of human brucellosis relies on three major approaches: (i) direct isolation by culture, (ii) PCR-based methods and (iii) indirect diagnosis by serological tests. Both isolation and PCR methods are laboratory intensive, require the right samples for isolation of *Brucella* and detection of *Brucella* genome and suffer from lower DSe [19]. Hence, serological tests outweigh the diagnosis of brucellosis in both humans and animals. In serological tests, specific titres in agglutination assays, cut-off values in ELISAs, or the presence of specific bands in LFAs are the hallmarks considered for diagnosis. However, these criteria are influenced by laboratory-dependent differences, clinical and epidemiological factors, and repeated exposure, which may affect the test interpretations [20,21]. The IgM antibody, which is readily detected by RBPT and SAT assays, initially dominates the humoral immune response to smooth *Brucella* species against the sLPS antigen in the first week. Among the IgG class of antibodies, IgG1 first develops around the second week followed by trace levels of IgG2 and even IgA, which peaks during the fourth week. In chronic brucellosis cases, IgM antibody decreases, allowing ELISAs to measure IgG and IgA [22]. Apart from the interplay of these immunoglobulins, non-agglutinating (incomplete) antibodies or antibodies to cytosolic proteins post treatment with antibiotics results in false negatives in chronic cases requiring additional tests like Coombs [23].

In this study, RBPT revealed an overall seropositivity of 7.67%, which decreased to 3.47% when confirmed with SAT. The results showed 57 (4.20%) cases having non-significant titres, implying greater exposure to *Brucella* in veterinary healthcare professionals, demanding periodical screening to assess the rise in titres. The significant titres were proportionately low (3.47%) affirming the use of two tests for accurate diagnosis of brucellosis without relying on only RBPT. These results align with prior research emphasizing the strong diagnostic performance of SAT, particularly in settings where high DSp is of paramount importance [24,25]. RBPT is qualitative in nature and manual interpretation of test results may vary among laboratory personnel. Also, the test has a tendency to overestimate results due to cross-reactivity and this is more significant for risk personnel as they are intermittently exposed to *Brucella* and other zoonotic pathogens. Hence, RBPT has to be confirmed with SAT titres for the interpretation of seropositivity in both animals and humans. The present study utilized RBPT and SAT as comparative reference assays because these tests remain the most widely used conventional serological methods for diagnosis of human brucellosis in India. However, both assays possess inherent limitations including cross-reactivity, subjective interpretation, and inability to differentiate active infection from previous exposure. Therefore, diagnostic performance estimates of LFAs should be interpreted considering the imperfect nature of these reference assays.

In the present study, SAT was performed with phenol saline which detects both IgM and IgG antibodies unlike 2ME-SAT which differentially detects IgG and IgM. In the laboratory setup, performing SAT and 2ME-SAT in tube formats for large sample volumes is tedious and time-consuming as SAT interpretation takes 18–24 h [8]. Handy, rapid and field-applicable tests are critical and hence the in-house developed LFA tests were utilized to look at the possibility of exploring its utility for differential and quick diagnosis of brucellosis in all diagnostic laboratory setups in India [26].

In the present study, 47 samples demonstrated significant SAT titres, whereas combined IgM and IgG LFAs detected 39 positives. The eight SAT-positive and LFA-negative samples were presumed to be due to lower SAT titres, suggesting antibody concentrations near the assay detection threshold. Such samples may represent early seroconversion, chronic low-grade exposure, past infections, nonspecific SAT cross-reactivity, or low immunoglobulin concentrations that were not detectable by LFAs. Conversely, a small number of LFA-positive but SAT-negative/non-significant titre samples were also observed, possibly reflecting persistence of IgG antibodies following past exposure or differences in antibody kinetics between assays. These discordant findings highlight the immunological dynamics inherent to brucellosis and hence no single serological assay can independently define infection status with certainty.

With the comparison of 1356 human serum samples among RBPT and IgM_LFA, the IgM_LFA showed a moderate DSe of 85.3% whereas other metrics were very good such as DSp, PPV, NPV, accuracy ~100% with a kappa value of 0.91. RBPT showed moderate DSe but had strong concordance with IgM_LFA, which is consistent with previous studies indicating the utility of RBPT in preliminary brucellosis testing [11,27]. Similarly, when RBPT was compared to IgG_LFA, the DSe was slightly lower at 82.69%, compared to DSp and PPV and NPV (100%). The overall accuracy of 98.67% and kappa value of 0.89 could be attributed to the dominance of IgM and IgG during acute/chronic or past infections which can be better detected by RBPT and IgG-based assays. As of now, RBPT cannot be ignored for preliminary testing of brucellosis in spite of its disadvantages.

The SAT recorded high DSe of 95.35% against IgM_LFA, with a perfect metric of >99% for DSp, PPV, NPV, accuracy and kappa value of 0.97. When compared with IgG LFA, SAT recorded again the high DSe of 96.55% similar to that of IgM_LFA coupled with a greater DSp, accuracy (>99%) and 0.96 kappa values. The near-perfect DSe observed with SAT indicates identification of actual infections, which is important for timely treatment. Similarly, high DSp facilitates undue diagnosis of healthy individuals, thereby causing anxiety in non-infected individuals [28]. The IgG_LFA (6.34%) showed higher positivity than IgM_LFA (2.95%), indicating that most infections in the study population may represent chronic or past exposure which is quite common in veterinary healthcare professionals [29]. These differential detections of IgM and IgG emphasize the need for such tests, especially for brucellosis [8,24,30]. Overall, IgM and IgG_LFA demonstrated excellent diagnostic performance against SAT, which is considered as a confirmatory test for brucellosis, further reinforcing its value in diagnosis.

Occupational risk analysis revealed higher seropositivity in paraveterinary staff (8.71%) than veterinary doctors (5.10%), and in males (8.3%) compared to females (4.02%), reflecting exposure patterns linked to daily handling of animals, reproductive materials, and high-risk interventions without adequate PPE [31]. District-wise variations in seropositivity observed in Figure 3 may be attributed to differences in bovine population density, intensity of occupational exposure, animal husbandry practices, awareness regarding zoonotic diseases, and compliance with personal protective measures. Districts with higher positivity rates may reflect increased exposure to infected animals and reproductive materials during routine veterinary activities. Age-stratified data indicated the highest seropositivity in individuals >55 years (9.09%), consistent with their long-term occupational exposure, as also reported in other regions [32]. These results highlight the importance of targeted surveillance, periodic screening, and protective measures for high-risk occupational groups to reduce brucellosis transmission. Paraveterinary personnel demonstrated higher positivity rates and a stronger regression coefficient (R^2^ = 87.89%) compared to veterinarians (R^2^ = 62.90%), indicating greater occupational exposure risk among paraveterinary staff and lack of compliance to PPE. The study population represented heterogeneous age groups, sexes, and professions with unknown previous brucellosis history, which may have influenced the observed diagnostic performance. The comparatively lower diagnostic sensitivity observed with RBPT may result from early stages of infection, prozone phenomenon, or low-affinity antibodies. Despite these limitations, LFAs address the critical need for rapid, field-based brucellosis diagnosis in endemic regions [8].

## 5. Conclusions

This study’s novelty lies in evaluating seroprevalence using multiple tests (RBPT, SAT, IgM_LFA, IgG_LFA) among veterinary personnel, revealing both acute and chronic exposure. Our findings support adopting IgM and IgG LFAs for their simplicity, rapid results, and ability to distinguish infection stages, aiding surveillance, monitoring and routine diagnosis while reducing dependence on conventional assays such as RBPT and SAT/2ME-SAT. This integrated approach enhances brucellosis detection and highlights the need for continued research to develop better serological and novel diagnostic tools. Clinical history/symptoms were not systematically documented during field screening camps, as the primary objective of the study was serological surveillance among occupationally exposed individuals. Therefore, seropositive cases identified in the study may represent both symptomatic and asymptomatic exposures. The limitation of the study is that the positive cases were not resampled or monitored post treatment because of the distant location of individuals. Similarly, the blood and other clinical samples were not assessed for isolation or PCR to define active infection. The personnel handled only cattle and buffaloes and hence exposure risks are presumed to be due to bovines only. The highest RBPT positivity was observed in Tumkur and Bangalore Rural districts and is attributed to the high density of bovine population, and these are also highest milk producing districts of the state. For differential comparison, 2ME-SAT was not performed due to shortage of serum samples volume. In our previous study, we compared IgM and IgG ELISA results, which similarly showed high DSe, DSp, and accuracy of >95%. Hence, we confined our study to evaluating field-applicable tests such as RBPT and SAT to LFAs. Another limitation of the study was the absence of bacterial isolation or molecular confirmation, which restricted differentiation between active infection and previous exposure. In addition, the use of RBPT and SAT as comparative reference standards may introduce classification bias because both assays have known limitations in sensitivity and specificity.

## Figures and Tables

**Figure 1:**
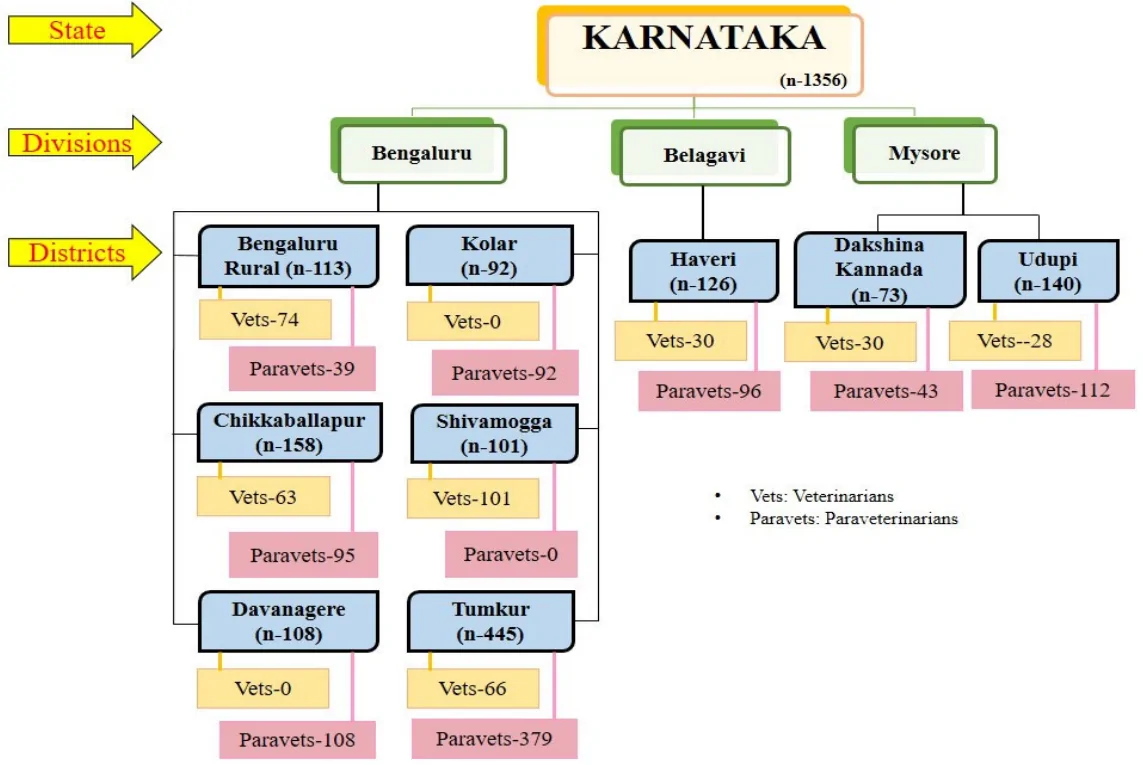
Sampling sites across different areas of Karnataka.

**Figure 2:**
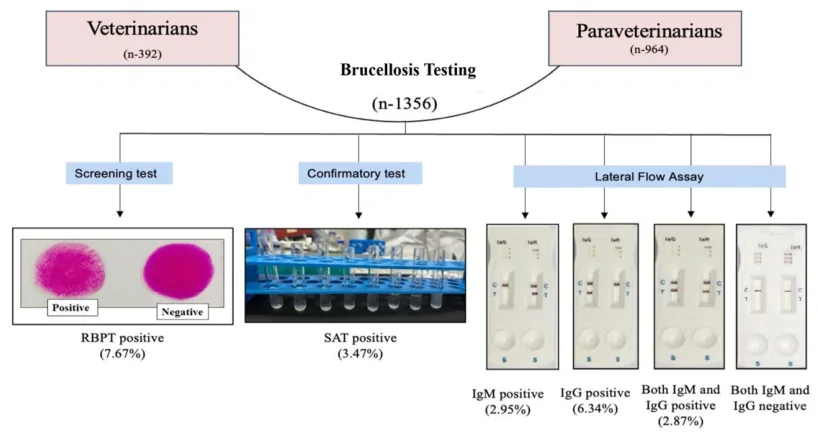
Schematic representation of serological tests employed for brucellosis testing of C = control line; T = test line; S = sample well.

**Figure 3:**
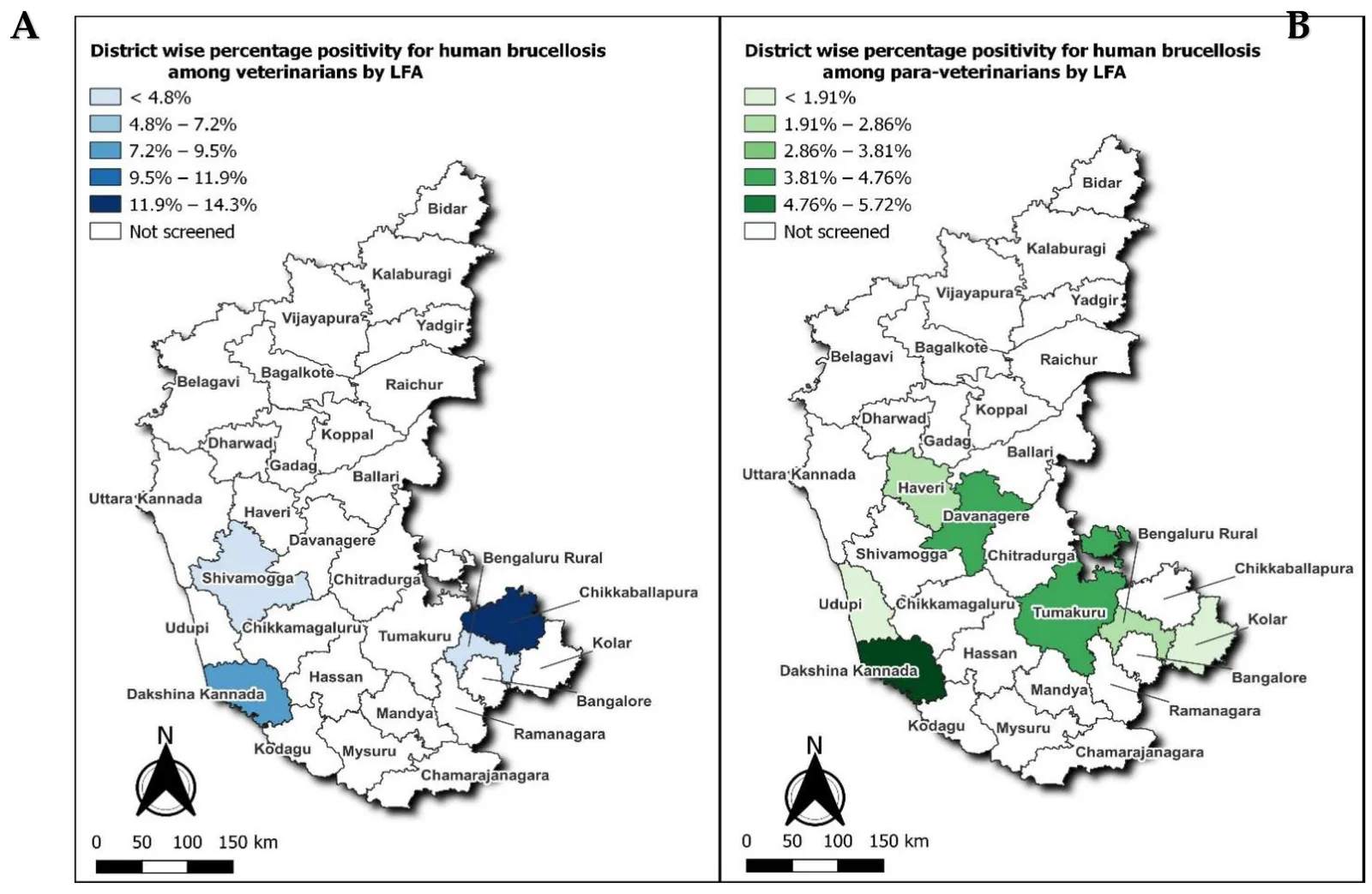
(**A**) District-wise percentage positivity among veterinarians; (**B**) district-wise percentage positivity among paraveterinary personnel.

**Figure 4:**
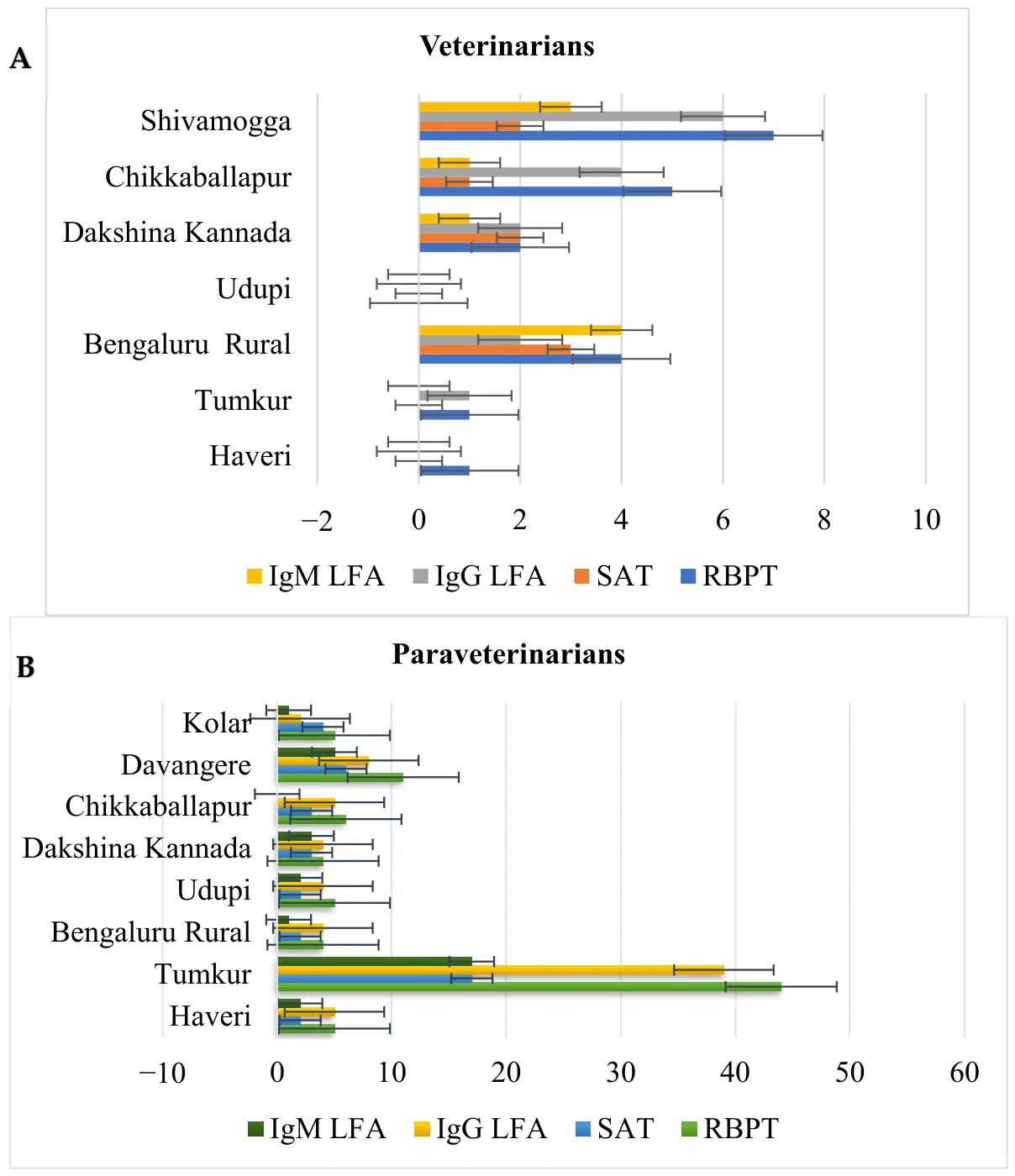
(**A**) Comparative test results for brucellosis in veterinarians in different districts of Karnataka. (**B**) Comparative test results for brucellosis in paraveterinarians in different districts of Karnataka.

**Figure 5:**
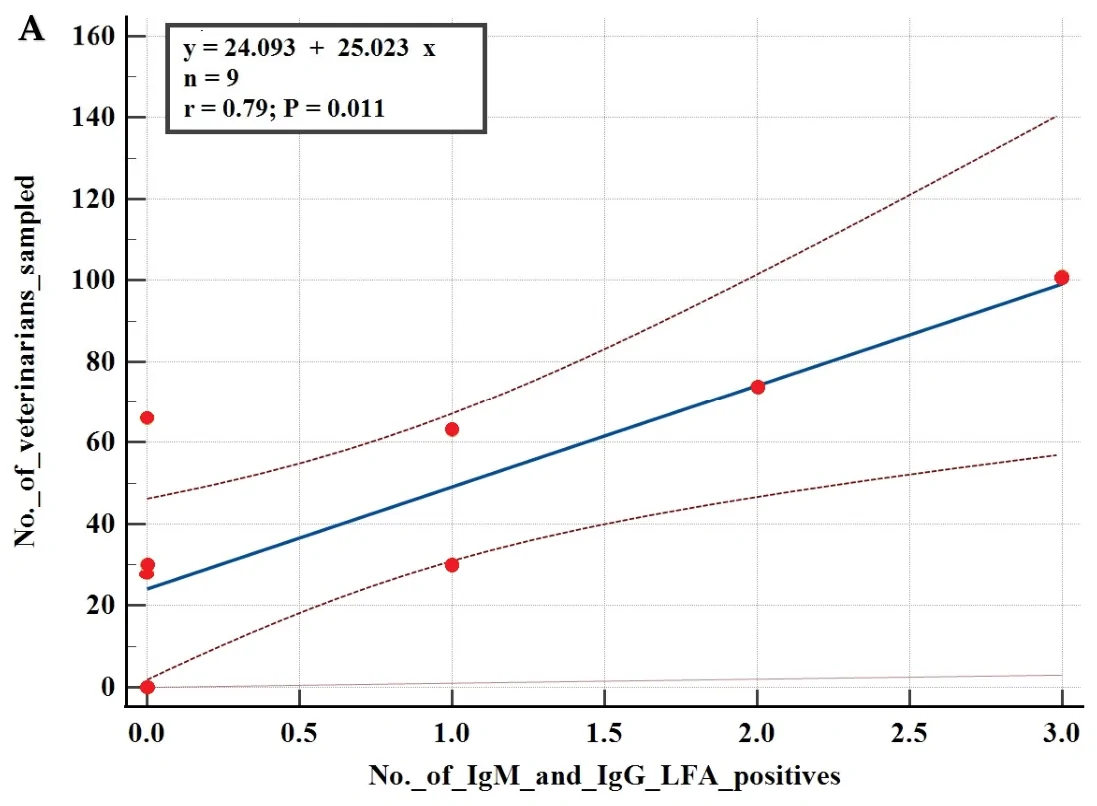

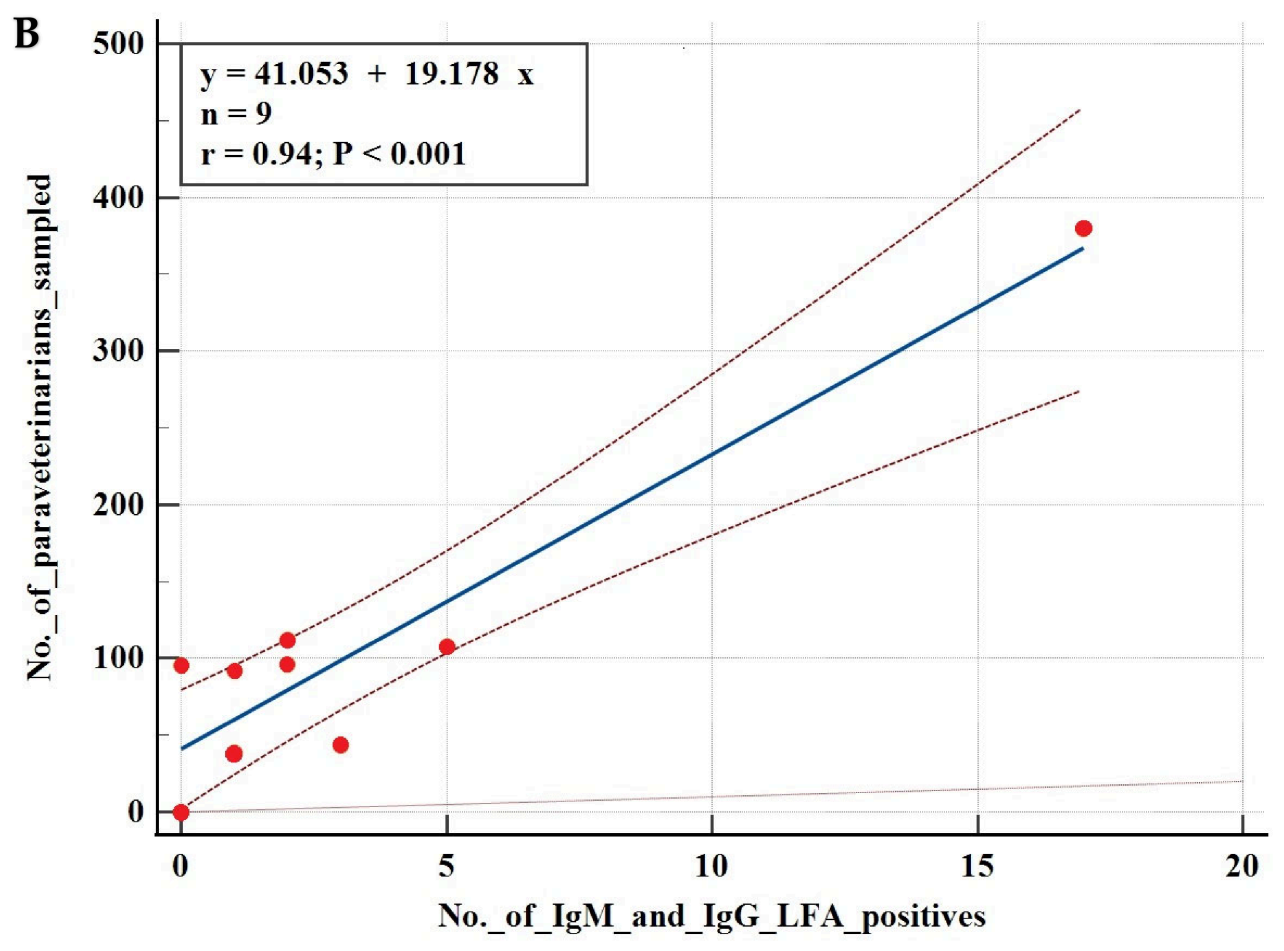
Regression analysis of IgM and IgG LFA positivity among (**A**) veterinarians and (**B**) paraveterinarians. The dotted line represents the linear regression trendline, while blue lines represent the regression fit, while red dots indicate observed positivity values.

**Table 1:** Comparative evaluation of IgM and IgG LFA with RBPT and SATs.

Sl. No	District-Wise	Occupation	No. of Samples	No. of Samples Positive by RBPT	Percent Positivity and CI at 95%	SAT Titre					No. of Samples Positive by SAT	Percent Positivity (CI at 95%)	Positive by IgG LFA	Percent Positivity (CI at 95%)	Positive by IgM LFA	Percent Positivity (CI at 95%)
						<160	160	320	640	˃640						
1	Haveri	Vets-30	126	1	3.33 (0.08–17.22)	1	-	-	-	-	-	-	-	-	-	-
		Paravets-96		5	5.21 (1.71–11.74)	3	-	1	1	-	2	2.08 (0.25–7.32)	5	5.21 (1.71–11.74)	2	2.08 (0.25–7.32)
2	Tumkur	Vets-66	445	1	1.52 (0.04–8.16)	1	-	-	-	-	-	-	1	1.52 (0.04–8.16)	-	-
		Paravets-379		44	11.61 (8.56–15.27)	27	7	7	3	-	17	4.49 (2.63–7.08)	39	10.29 (7.42–13.80)	17	4.49 (2.63–7.08)
3	Bengaluru Rural	Vets-74	113	4	5.41 (1.49–13.27)	1	2	-	1	-	3	4.05 (0.84–11.39)	2	-	4	5.41 (1.49–13.27)
		Paravets-39		4	10.26 (2.87–24.22)	2	1	1	-	-	2	5.13 (0.63–17.32)	4	10.26 (2.87–24.22)	1	2.56 (0.06–13.48)
4	Udupi	Vets-28	140	-	-	-	-	-	-	-	-	-	-	-	-	-
		Paravets-112		5	4.46 (1.47–10.11)	3	2	-	-	-	2	1.79 (0.22–6.30)	4	3.57 (0.98–8.89)	2	1.79 (0.22–6.30)
5	Dakshina Kannada	Vets-30	73	2	6.67 (0.82–22.07)	-	2	-	-	-	2	6.67 (0.82–22.07)	2	6.67 (0.82–22.07)	1	3.33 (0.08–17.22)
		Paravets-43		4	9.30 (2.59–22.14)	1	2	1	-	-	3	6.98 (1.46–19.06)	4	9.30 (2.59–22.14)	3	6.98 (1.46–19.06)
6	Chikkaballapur	Vets-63	158	5	7.94 (2.63–17.56)	4	1	-	-	-	1	1.59 (0.04–8.53)	4	6.35 (1.76–15.47)	1	1.59 (0.04–8.53)
		Paravets-95		6	6.32 (2.35–13.24)	3	-	1	1	1	3	3.16 (0.66–8.95)	5	5.26 (1.73–11.86)	-	-
7	Davanagere	Paravets-108	108	11	10.19 (5.19–17.49)	5	1	2	3	-	6	5.56 (2.07–11.70)	8	7.41 (3.25–14.07)	5	4.63
8	Shivamogga	Vets-101	101	7	6.93 (2.83–13.76)	5	2	-	-	-	2	1.98 (0.24–6.97)	6	5.94 (2.21–12.48)	3	2.97 (0.62–8.44)
9	Kolar	Paravets-92	92	5	5.43 (1.79–12.23)	1	1	-	1	2	4	4.35 (1.20–10.76)	2	2.17 (0.26–7.63)	1	1.09 (0.03–5.91)
Total		Vets-392	1356	20	5.10 (3.14–7.77)	12	7	-	1	-	8	2.04 (0.89–3.98)	15	3.83 (2.16–6.23)	9	2.30 (1.06–4.31)
		Paravets-964		84	8.71 (7.01–10.67)	45	14	13	9	3	39	4.05 (2.89–5.49)	71	7.37 (5.80–9.20)	31	3.22 (2.20–4.53)
		Cumulative total		104	7.67 (6.31–9.22)						47	3.47 (2.56–4.58)	86	6.34 (5.10–7.77)	40	2.95 (2.12–4.00)

**Table 2:** Diagnostic performance of IgM and IgG LFA in comparison to RBPT and SAT.

Test Criteria	Sensitivity	Specificity	PPV	NPV	Accuracy	Kappa
RBPT v/s IgM LFA	85.3 (74.6–92.7)	100 (99.2–100)	100 (95.80–100.00)	99.55 (99.0–99.8)	99.56 (98.6–99.6)	0.91 (0.85–0.96)
SAT v/s IgM LFA	95.35 (84.2–99.4) *	100 (99.2–100)	100 (99.2–100)	99.84 (99.1–99.9)	99.85 (98.8–99.9)	0.97 (0.94–1)
RBPT v/s IgG LFA	82.69 (74.03–89.41)	100 (99.71–100)	100 (95.80–100.00)	98.58 (97.8–99.1)	98.67 (97.9–99.2)	0.89 (0.85–0.94)
SAT v/s IgG LFA	96.55 (88.1–99.6)	99.54 (98.36–99.9)	91.76 (73.6–97.8)	99.82 (99.3–99.9)	99.41 (98.2–99.8)	0.96 (0.92–0.99)

PPV, positive predictive value; NPV, negative predictive value; * values in parenthesis are at 95% confidence interval; IgG, immunoglobulin G; IgM, immunoglobulin M; LFA, lateral flow assay; RBPT, Rose Bengal Plate Test.

**Table 3:** Analysis of human brucellosis with respect to sex, age and profession.

Host Factors		Total Number of Samples	No. of Samples Positive	Percent Positivity and CI at 95%	χ^2^ (*p*-Value)	Odds Ratio
Sex	Male	1157 (85.32)	96	8.30 (6.77–10.04)	3.80 (0.0051)	Ref.
	Female	199 (14.67)	8	4.02 (2.44–9.05)		2.16
	Total	1356	104	7.67 (6.31–9.22)		-
Age	<25.0	35 (2.58)	0	0	4.02 (0.403)	0.00
	25.1–35.0	352 (25.95)	24	6.82 (4.42–9.98)		Ref.
	35.1–45.0	302 (22.27)	24	7.95 (5.16–11.59)		1.17
	45.1–55.0	447 (32.96)	36	8.05 (5.70–10.98)		1.21
	>55.1	220 (16.22)	20	9.09 (5.64–13.69)		1.37
	Total	1356	104	7.67 (6.31–9.22)		-
Profession	Veterinary Doctors	392 (28.90)	20	5.10 (3.14–7.77)	4.89 (0.0269) *	0.55
	Paravets	964 (71.09)	84	8.71 (7.20–10.90)		Ref.
	Total	1356	104	7.67 (6.31–9.22)		-

* *p* < 0.05; values in parenthesis represent percentage.

**Table 4:** Regression analysis of IgM and IgG LFA in veterinarians and paraveterinarians.

Sl. No.	Districts	No. of Veterinarians Sampled	No. of IgM and IgG LFA Positives	No. of Paraveterinarians Sampled	No. of IgM and IgG LFA Positives
1	Haveri	30	0	96	2
2	Tumkur	66	0	379	17
3	Bangalore Rural	74	2	39	1
4	Udupi	28	0	112	2
5	Dakshina Kannada	30	1	43	3
6	Chikkaballapur	63	1	95	0
7	Davanagere	0	0	108	5
8	Shivamogga	101	3	0	0
9	Kolar	0	0	92	1
	Total	392	7 (1.78%)	964	31 (3.21%)
	R^2^	62.90		87.89	
	*p*-value	0.0107		0.0001	

## Data Availability

The original contributions presented in this study are included in the Article. Further inquiries can be directed to the corresponding author.

## References

[1] Mohseni A.H., Shahin K., Bayani M., Oskouee M.A. (2017). The Molecular Detection and Prevalence of *Brucella spp.* in Raw Milk from Different Animal Species in Iran Using PCR Assay. Vet. World.

[2] Corbel M.J. (1997). Brucellosis: An Overview. Emerg. Infect. Dis..

[3] Boschiroli M.L., Ouahrani-Bettache S., Foulongne V., Michaux-Charachon S., Bourg G., Allardet-Servent A., Cazevieille C., Liautard J.P., Ramuz M., O’Callaghan D. (2002). The *Brucella suis* VirB Operon Is Induced Intracellularly in Macrophages. Proc. Natl. Acad. Sci. USA.

[4] Wang Y., Xu C., Zhang S., Wang Z., Zhu Y., Yuan J. (2018). Temporal Trends Analysis of Human Brucellosis Incidence in Mainland China from 2004 to 2018. Sci. Rep..

[5] Mantur B.G., Amarnath S.K. (2008). Brucellosis in India–A Review. J. Biosci..

[6] Singh B.B., Khatkar M.S., Aulakh R.S., Gill J.P.S., Dhand N.K. (2018). Estimation of the Health and Economic Burden of Human Brucellosis in India. Prev. Vet. Med..

[7] Young E.J. (1983). Human Brucellosis. Rev. Infect. Dis..

[8] Shome R., Patra S., Sahib M.M., Shanmugam G., Dubey S., Skariah S., Shamshad S., Barman N.N., Bora D.P., Shome A. (2023). Evaluation of an In-House IgM/IgG Lateral Flow Assay for Serodiagnosis of Human Brucellosis. Indian J. Med. Microbiol..

[9] Panjarathinam R., Jhala C.I. (1986). Brucellosis in Gujarat State. Indian J. Pathol. Microbiol..

[10] Thakur S.D., Thapliyal D.C. (2002). Seroprevalence of Brucellosis in Man. J. Commun. Dis..

[11] Mantur B.G., Biradar M.S., Bidri R.C., Mulimani M.S., Veerappa K., Kariholu P., Patil S.B., Mangalgi S.S. (2006). Protean Clinical Manifestations and Diagnostic Challenges of Human Brucellosis in Adults: 16 Years’ Experience in an Endemic Area. J. Med. Microbiol..

[12] Agasthya A.S., Isloor S., Prabhudas K. (2007). Brucellosis in High-Risk Group Individuals. Indian J. Med. Microbiol..

[13] Prakash P., Bhansali S., Gupta E., Kothari D., Mathur A., Ambuwani S. (2012). Epidemiology of Brucellosis in High-Risk Group and PUO Patients of Western Rajasthan. Natl. J. Community Med..

[14] Shome R., Padmashree B.S., Triveni K., Krithiga N., Sahay S., Shome B.R., Chandrashekar N., Rahman H. (2015). Multipronged Diagnostic Approaches for Monitoring the Treatment of *Brucella abortus* Infected Patient: A Case Report. Asian Pac. J. Trop. Dis..

[15] Wright A.E., Smith F. (1887). On the Application of the Serum Test to the Differential Diagnosis of Typhoid and Malta Fever. Lancet.

[16] Alton G.G., Jones L.M., Angus R.D., Verger J.M. (1988). Techniques for the Brucellosis Laboratory.

[17] Adetunji S.A., Ramirez G., Foster M.J., Arenas-Gamboa A.M. (2019). A Systematic Review and Meta-Analysis of the Prevalence of Osteoarticular Brucellosis. PLoS Negl. Trop. Dis..

[18] Zheng R., Xie S., Lu X., Sun L., Zhou Y., Zhang Y., Wang K. (2018). A Systematic Review and Meta-Analysis of Epidemiology and Clinical Manifestations of Human Brucellosis in China. Biomed. Res. Int..

[19] Dean A.S., Crump L., Greter H., Hattendorf J., Schelling E., Zinsstag J. (2012). Clinical Manifestations of Human Brucellosis: A Systematic Review and Meta-Analysis. PLoS Negl. Trop. Dis..

[20] Abo-Shehada M.N., Odeh J.S., Abu-Essud M., Abuharfeil N. (1996). Seroprevalence of Brucellosis among High-Risk People in Northern Jordan. Int. J. Epidemiol..

[21] Ariza J., Pellicer T., Pallarés R., Foz A., Gudiol F. (1992). Specific Antibody Profile in Human Brucellosis. Clin. Infect. Dis..

[22] Di Bonaventura G., Angeletti S., Ianni A., Petitti T., Gherardi G. (2021). Microbiological Laboratory Diagnosis of Human Brucellosis: An Overview. Pathogens.

[23] Bowden R.A., Racaro G.C., Baldi P.C. (1999). Effect of Early Antibiotic Treatment on the Antibody Response to Cytoplasmic Proteins of *Brucella melitensis* in Mice. Clin. Diagn. Lab. Immunol..

[24] Godfroid J., Nielsen K., Saegerman C. (2010). Diagnosis of Brucellosis in Livestock and Wildlife. Croat. Med. J..

[25] Gwida M., Al Dahouk S., Melzer F., Rösler U., Neubauer H., Tomaso H. (2010). Brucellosis-Regionally Emerging Zoonotic Disease?. Croat. Med. J..

[26] Ge L., Wang D., Lian F., Zhao J., Wang Y., Zhao Y., Zhang L., Wang J., Song X., Li J. (2021). Lateral Flow Immunoassay for Visible Detection of Human Brucellosis Based on Blue Silica Nanoparticles. Front. Vet. Sci..

[27] World Organisation for Animal Health (OIE) (2018). Brucellosis (*Brucella abortus*, *B. melitensis* and *B. suis*). Terrestrial Manual.

[28] Yagupsky P., Morata P., Colmenero J.D. (2019). Laboratory Diagnosis of Human Brucellosis. Clin. Microbiol. Rev..

[29] Nour B., Tawor B., Gebril A., Mohamedani A.A., Saeed O.K. (2015). Evaluation of Simple and Rapid Specific IgM/IgG Flow Assay and Some Methods Used in Diagnosis of Brucellosis in Gezira and Blue Nile States, Sudan. Clin. Microbiol..

[30] Zhang N., Fang X., Zhou W., Tan Z., Liang S., Wang X., Hu J.L., Bao C.J., Liu W.D. (2023). Epidemiological Characteristics and Temporal-Spatial Clustering Analysis on Human Brucellosis in Jiangsu Province, 2006–2021. Sci. Rep..

[31] Loubet P., Magnan C., Salipante F., Pastre T., Keriel A., O’Callaghan D., Sotto A., Lavigne J.P. (2024). Diagnosis of Brucellosis: Combining Tests to Improve Performance. PLoS Negl. Trop. Dis..

[32] Shome R., Kalleshamurthy K., Shankaranarayana P.B., Giribattanvar P., Chandrashekar N., Mohandoss N., Shome B.R., Kumar A., Barbuddhe S.B., Rahman H. (2017). Prevalence and Risk Factors of Brucellosis among Veterinary Health Care Professionals. Pathog. Glob. Health.

